# Potential value of home square-stepping exercises for inactive older adults: an exploratory case study

**DOI:** 10.1186/s12877-021-02712-x

**Published:** 2022-01-03

**Authors:** Marcus A. Lees, Jonathon Edwards, Jamie E. McCain, Danielle R. Bouchard

**Affiliations:** 1 Faculty of Kinesiology, University of New Brunswick, 90 MacKay Dr, Fredericton, NB E3B 5A3 Canada; 2 Cardiometabolic Exercise & Lifestyle Laboratory, Faculty of Kinesiology, University of New Brunswick, 90 Mackay Drive, Room 105, Fredericton, NB E3B 5A3 Canada

**Keywords:** Aging, Physical function, Inactivity, Case study, Geriatrics, Successful aging

## Abstract

**Background:**

Most older adults do not engage in regular physical activity. However, more research on options to partake in regular exercise in this population by reducing barriers and enhancing enablers while still reaching benefits is needed.

**Methods:**

Using embedded mixed methods, 10 inactive older adults over the age of 65 completed a 3-week square-stepping exercise intervention to help overcome the initial barriers and activate initial enablers to perform regular exercise. Physical activity level was tracked at home with a pedometer using median steps/day over seven days for pre-post measure. Aerobic intensity while doing square-stepping exercises was quantified via a heart rate monitor in a supervised session. Each participant had an interview asking about barriers and enablers to regular exercise and if the intervention could modify any. Based on initial physical activity a framework matrix was used to pull potential barriers to compare, contrast, and search for patterns between participants with lower and higher initial physical activity levels.

**Results:**

The 3-week square-stepping exercise intervention helped participants overcome barriers such as being uncomfortable in a fitness facility and body image and activate enablers such as the use of home equipment and convenience. The median total steps/day increased by 12% (*p* = 0.02), and a moderate-intensity level was reached by 80% of the sample participants when performing the square stepping exercise during a supervised session. Common barriers such as having a suitable program, hard to keep the intensity were reported by participants regardless of the initial physical activity level.

**Conclusion:**

Regardless of initial physical activity level, inactive older adults can increase physical activity level at the recommended intensity and overcome common barriers to exercise when performing square-stepping exercises, especially for those intimidated by a fitness facility setting and those concerned with their body image. A longer intervention including more participants using the square-stepping exercises is required to understand if square-stepping exercises can increase the proportion of older adults exercising regularly.

**Supplementary information:**

The online version contains supplementary material available at 10.1186/s12877-021-02712-x.

## Background

The proportion of the world’s population over 60 years is predicted to almost double between 2015 and 2050 as older adults will outnumber young children due to population aging [1]. Most countries are aiming to increase the number of older adults who remain in an independent living situation. It is important for older adults to stay physically active to remain independent. Thus, exercise is an option for being physically active as it reduces the risk of chronic conditions, frailty, hospital stays, and mortality [2]. Adults 65 years or older are recommended to do at least 150 min of moderate-intensity to vigorous-intensity aerobic physical activity per week [3]. Moderate intensity is typically the minimum intensity recommended during aerobic exercise by many international and national agencies to optimize health and functional benefits [3, 4]. Unfortunately, less than 15% of older adults meet the recommendations when exercise is measured objectively [5]. It is thus important to identify strategies that lead to regular exercise in this population, taking into consideration the barriers and enablers that will motivate this target population to engage.

Previous research has identified several barriers and enablers to regularly engaging in physical activities for individuals over the age of 65. Barriers to exercise have included lack of time [6], pain/injury [7–10], and lack of motivation [8, 9]. Conversely, some enablers identified in previous research have included perception of health benefits [8–12], social support [8–11], and enjoyment [8–11, 13]. Home-based exercise can help encourage lifestyle changes that may help older adults overcome barriers to regular physical activity [14].

Home-based exercise offers the opportunity to complete exercise every day using a flexible schedule at a low cost [15]. Square-Stepping Exercise is an example of a home-based aerobic exercise [16]. Shigematsu and Okura [17] developed this exercise in Japan and it was deemed suitable for home settings [16]. The square-stepping exercises are performed on a thin felt mat that is two meters in length and divided into small squares. It is an unobtrusive piece of equipment to be used in the home.

In general, taking multidirectional steps in a pattern was shown to enhance motor learning, balance, and memory that could potentially prevent falls [18]. Using square-stepping exercises/patterns can be developed to fit different purposes, settings, and populations [18]. Square-stepping exercises were found to be safe and acceptable, especially for lower-body functional fitness and it may be recommended to older adults due to its low cost and effectiveness [19]. Research has shown that the square-stepping exercises are effective in strength and balance training [19] and can improve cognitive impairment as a result of changing stepping patterns [19]. The exercise could also be more effective than walking programs in reducing falls [20]. Despite these results, the intensity reached while performing the square-stepping exercises is unknown.

After reviewing the literature on adverse consequences of physical inactivity, it was determined that a home-based exercise intervention using square-stepping exercises could promote accessible physical activity outside of fitness facilities. Therefore, the purpose of this study was to examine whether a 3-week intervention using square-stepping exercises could 1- overcome barriers and activate enablers to exercise identified prior to the intervention using semi-structured interviews, 2- increase physical activity levels (via a change in weekly median steps per day), 3- increase the intensity above moderate (via HR monitor) when performing a supervised session and 4- Explore participants’ characteristics and measured outcomes of the square stepping intervention in relation to their perceived barriers post-intervention. The primary research question was: Can square-stepping exercises be used to overcome perceived barriers to exercise and activate enablers to exercise? While, the secondary research question was: Can the square-stepping exercise increase physical activity level and reach the recommended intensity when performing a supervised session? The final research question was: How do participants’ characteristics and measured outcomes following a square stepping exercise intervention relate to their reported barriers post-intervention?

## Methods

### Study design

This research is foundational and exploratory as literature on square-stepping exercises is limited. Therefore, a single case study design was applied to address the research questions. Case study research “explores real-life, contemporary bounded system (a case) or multiple bounded systems (cases) over time, through a detailed, in-depth analysis using multiple sources of information” [21]. The use of data from multiple sources aid in identifying case themes and descriptions [21]. This approach was deemed to be appropriate as it provides “an empirical inquiry that investigates a contemporary phenomenon (the “case”) within its real-world context, especially when the boundaries between phenomenon and context are not clearly evident” [22]. Thus, the case in this study is understood to examine older adults exercising in their homes using square-stepping exercises to overcome the barriers associated with exercising while enhancing enablers that motivate these individuals to engage in physical activity.

Mixed methods research and case study research combined offer a unique methodological advantage for those researchers looking to address complex problems and issues, according to Plano Clark, Foote, and Walton [23]. Thus, this current research used both mixed methods and a case study research design to address the research questions presented above. More specifically, Ivankova and Creswell [24] described embedded mixed methods as one data set providing a supportive and secondary role in a study based primarily on the other type of data. For this study, the quantitative data were embedded into the qualitative approach, which makes the qualitative strand primary. We specifically measured any changes in physical activity level (e.g., intensity, median steps per day), while gaining insight into the impact on the sample’s ability to effectively execute the square-stepping exercises at the recommended intensity level. This project was approved by a Research Ethics Board at the authors' academic institution before starting the recruitment of participants.

### Participants

Purposeful and convenience sampling was used to identify information-rich cases that provided information about issues of central importance [25]. Therefore, the selected sample was purposefully inactive older adults who consented to participate in the home-based square stepping exercise intervention called Home-Steps. As well, the geographical location of the study attracted many participants that had post-secondary education and high-income levels. This location was also convenient for the researchers. Participants recruited were from a small city in Canada through advertising by flyers in local public facilities (e.g., gyms, exercise, and churches), internet (e.g., Facebook), and different newspapers.

Inclusion criteria for the participants were the following: Age 65–80; not receiving any kind of home care services (formal or informal); being cleared to exercise using the Get Active Questionnaire [26]; having a resting heart rate < 99 and blood pressure < 160/90; and, recording a median total steps/day < 10,000 measured over seven days via a pedometer during week 1 (Steps Count, StepRX) [27].

Although older adults are typically classified as being 65 years or older with no cut-off age [28], this study targeted older adults that are still mobile enough to do the exercise safely. The Get Active Questionnaire developed by the Canadian Society for Exercise Physiology (CSEP) is an evidence-based self-administered tool that was used to safely encourage and screen-in participants to become more physically active [29, 30]. The questionnaire assisted in making informed decisions on whether participants should seek further advice from a qualified professional before participating in the exercise intervention. Participants had to be taking less than < 10,000 steps/day as a weekly median to participate as this is normally recommended for adults [27].

### Measures

#### Qualitative measures

Qualitative measures include semi-structured interviews to explore participants’ barriers and enablers of the Home Steps intervention. After completing the Home Steps intervention, each participant was involved in a 30 to 60-min interview. Interviews were done face-to-face. Semi-structured interviews with open-ended questions were used to allow participants to respond freely and provide descriptions of their perceptions in more detail [31]. A total of 14 open-ended questions were asked (see Additional File 1 for the interview guide). The interview guide was created based on previous literature and practical experience. To ensure the trustworthiness of the interview guide, a pilot test was conducted to determine two parts of the research design: 1) to determine the appropriate exercises for the home-based exercise intervention; and, 2) to test the interview questions. During this process, some interview questions were modified to enhance the richness of the data that was going to be collected.

By using open-ended questions new ideas emerged that were not identified in the literature. The majority of the questions posed related to personal experiences of becoming more physically active with the intervention. All interviews were audio-recorded and transcribed with participant permission by the primary researcher. The primary researcher reviewed the audio-recorded interviews twice to ensure that they were transcribed accurately to reproduce all spoken words. Participants’ names were changed to ensure confidentiality. Data saturation is often used to determine when to end conducting interviews in qualitative research [32]. Data saturation is understood to be the point where the primary researcher is not receiving any new information from the interviews. After analyzing the data of 10 participants, it was determined that saturation was reached.

#### Quantitative measures

Quantitative measures include tracking total average steps per day for a week via a Step RX pedometer (Steps Count, StepRX, Ontario, Canada) [33]. A minimum of four consecutive days of wear-time had to be obtained to be considered as a valid measure [34]. The intensity reached while performing the pattern of the square stepping session was measured via HR monitor Polar V800 [35]. Heart rate (HR) was registered via the HR sensor (Polar H7, Woodbury, New York, USA). To estimate the exercise intensity, HR reserve (HRR) was calculated by (max HR-resting HR) *0.40 + resting HR; max HR was calculated by 220-age. The median HR while performing home square-stepping exercise pattern was recorded during minutes 5 to 10. To reach moderate intensity, 40% of HRR had to be achieved [36]. Resting Heart Rate (RHR) and Blood Pressure (BP) was measured using an automatic blood pressure cuff (Omron, blood pressure monitor, Kyoto, Japan) for safety purposes. Height and body weight were measured using a stadiometer (Seca, stadiometer, Hamburg Germany). The Four Senior Fitness Tests (SFT) used were the 2-min step test (endurance), 8 foot up and go (agility), and squat to chair (lower body strength), and arm curl (upper body strength) to measure physical function [37]. A description, the validity, and reliability of tests is available within the Senior Fitness Test Manual by Rikli and Jones [38]. The unipedal test was used to measure balance which is a validated test [39]. Participants self-reported a level of importance (e.g., 1: not very important to 10: very important) on 25 barriers to exercise with a maximum score of 250 made through the CSEP [26]. Participants answered additional demographic questions regarding their ethnicity, level of education obtained, age, gender, and personal income.

### Exercise intervention

**Fig. 1:**
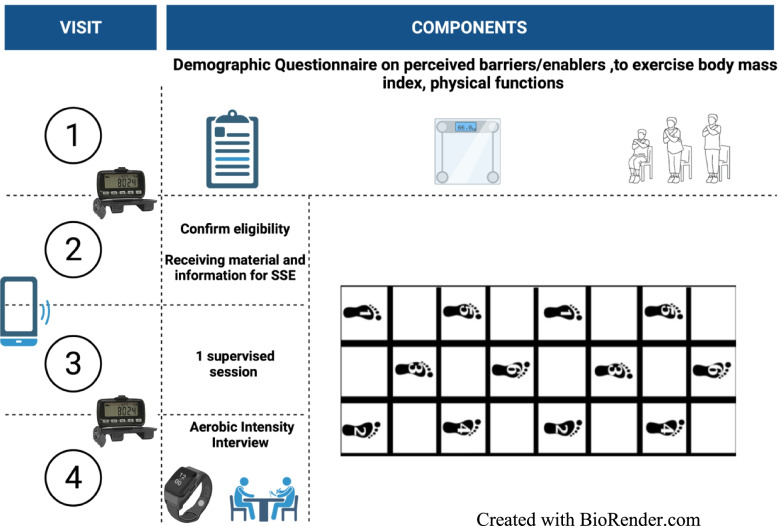
Study Design

The 3-week home square-stepping exercise intervention called *Home Steps* is intended to increase physical activity level to 150 min per week according to the physical activity guidelines. Participants were asked to do a stepping sequence that only included forward and oblique steps (see Fig. 1). This pattern only had 12 steps compared to more advanced patterns that had 32 steps. Typically, the participants stepped forward on the mat and circled around the mat to complete the stepping sequence again. However, they could have chosen to go back and forth to their starting point as well. Participants were asked to complete the pattern repeatedly in 10-min bouts to try to reach the goal of 150 min a week.

A square stepping mat that was 200 × 100 cm and divided into 32 small squares (25 × 25 cm) was provided to each participant. The mat was smaller compared to the mat used in the successful study conducted by Shigematsu and Tomohiro [17]. Using a smaller mat helped ensure that it would fit in the participant’s homes. The mat was handmade with scotch tape and a Bristol board with the squares drawn out using a black marker. This cost-effective approach enabled the mats used in *this* intervention to be made for less than $5.00 CDN ($3.98 US).

The researcher met four times with the participants individually in a room on a university campus. The room mimicked a home setting where the only requirement was a flat floor. Beyond the in-person visits, the study participants were responsible for completing the remaining part of the home square stepping exercises by themselves. The following visits are further explained below.

#### Visit One (60-min)

Written informed consent was obtained from all the study participants allowing them to participate and allowing the researcher to publish patient information. Then, participants performed physical function tests, had their anthropometrics measured, and had to rank barriers to exercise to determine baseline data. Additionally, RHR and BP were also measured, and demographic questions were asked. Participants were also given a Step RX pedometer (Steps Count, StepRX, Ontario, Canada) to wear to measure total steps for seven consecutive days pre-intervention to determine their physical activity status [34].

#### Visit Two (45-min)

During the second visit, participants returned the Step RX pedometer and the research team confirmed their eligibility criteria. Eligible participants were taught the square stepping exercise and were given a square stepping exercise mat to bring home. They had a weekly goal of completing three sessions of 50 min per day.

#### Visit Three (45-min)

Prior to this visit, a phone call was made to each participant to answer any questions about the exercise intervention. Then they visited to perform a session under supervision. Participants were then given a Step RX pedometer again to wear for another week. The intention of the pedometer would be to measure the square stepping exercise completed at home.

#### Visit Four (90-min)

Participants returned the Step RX pedometer and completed a warm-up (participants performing the stepping sequence five times) before completing a 10-min session of the square stepping exercise to quantify the intensity when performing the prescribed square stepping pattern. Heart rate (HR) was registered via the HR sensor for a 10-min bout of the square stepping exercise (Polar H7, Woodbury, New York, USA). No feedback from the staff was made. Afterward, participants completed a 30 to 60-min interview. See Fig. 1 for more information.

### Data analysis

#### Qualitative

The interviews were analyzed using the framework method. The approach originated in large-scale social policy research but has become more popular in health research [40]. The framework method was developed by Ritchie and Lewis and was used to analyze data for a matrix output [41]. The framework method consisted of five stages [42]:

1. Familiarization – Interview transcriptions were reviewed while listening to the corresponding audio file to ensure content accuracy.
2. Thematic identification – Leximancer 4. was used to initially determine themes and concepts identified from the interviews. Leximancer is a computer qualitative data analysis software that provides a form of automated handling of qualitative data based on the statistical properties of text [43]. Through Leximancer, the software can identify the keywords used in the text that continue to appear and highlight the semantic relationships and proximity between the identified words. The transcripts were read several times before marking reoccurring phrases and keywords to make themes.
3. Indexing or coding – Following thematic identification, the researchers conducted a manual review, where the interview transcripts were coded for themes and compared with the results from Leximancer as a means of ensuring the trustworthiness of the coding process.
4. Charting—QSR NVivo 12 was used to identify and organize quotes into corresponding themes from which charts were created that provide detailed summaries of the results of the interview process. NVivo is a similar computer software tool to that of Leximancer; however, NVivo enables the researcher to manually code different types of documents. Once the charts were completed, two members of the research team reviewed the charts in correlation with the data collected to ensure accuracy in the data analysis process. Mapping and interpretation—Explained in the Mixed Methods section below.Triangulation and debriefing sessions were two strategies that were used to enhance trustworthiness [21]. Triangulation, common in using case studies, is when multiple sources of data are used to augment one another to ensure the accuracy of the data. Debriefing sessions were completed at the end of the intervention with the participants. Since quantitative data was embedded into this case study, different sources of evidence were used to explore if this intervention provided benefits by doing frequent debriefing sessions with an experienced researcher to discuss alternative approaches, draw attention to potential flaws, and act as a sounding board to test and/or develop ideas. According to Yin (2003) [22], investigators must be challenged on their case study assumptions/interpretations to adequately represent different perspectives.

#### Quantitative

Most values are reported as individual data and median to describe the sample. The Wilcoxon Signed Ranks Test was used to quantify the change pre-post on median steps daily for the sample. All variables and the analysis were treated in SPSS version 22.

#### Mix-methods

The last two steps of the framework method interpreted the quantitative and qualitative results [41]. NVivo was used in the fourth step to streamline the qualitative and mixed methods data by organizing and managing data from different sources [44]. Therefore, NVivo was used to manually code the qualitative and quantitative data and create a chart to compare the data. During the last stage, mapping and interpretation, the quantitative and qualitative results were combined.

Barriers and enablers were cross-referenced with data collected before or after the intervention as means of interpreting the findings. This was done to interpret and discuss specific cases, perceptions, experiences, patterns, and connections in search of explanation and meaning.

The analysis was shown to be suitable for both qualitative and quantitative researchers and provided a spreadsheet format and clear steps to follow for qualitative exploration [40]. The matrix output offered interconnected data for charting quantitative and qualitative data, which included rows (cases), columns (codes), and ‘cells’ that summarized this data. However, caution was recommended as data sets should cover similar topics and be overseen by an experienced researcher. The findings are discussed through the lens of the social-ecological model, which is known to fit well in the domain of physical activity [45].

## Results

### Quantitative data

Demographic and characteristic information on participants is presented in Table 1.

**Table 1:** Information on Participants

	Median	1	2	3	4	5	6	7	8	9	10
Age (years)	70.7	66	74	76	70	67	76	68	75	65	71
Body mass index (kg/m^2^)	31.6	32.8	30.4	28.6	32.1	28.4	33.1	31.0	34.1	29.9	26.8
Sex		M	M	M	M	F	F	F	F	F	F
Education Level		HS	U	U	HS	U	U	D	D	HS	D
Personal Income ($)		3	4	5	2	5	5	1	2	0	4

Sex: *M* Male, *F* Female

Education Level *HS* High School, *D* Diploma, *U* University

Personal incomes: 0 Less than 20,000, 1:20,000 to 34,999, 2: 35,000 to 49,999, 3: 50,000 to 74,999, 4: 75,000 to 99,999

The sample included 10 participants, of whom six were female. The median age was 71 years with a median body mass index (BMI) of 31.6 kg/m^2^. All participants completed at least their high school; four participants obtained a university degree. Five participants had personal incomes above $35,000/year. Physical function measurements (Table 2) showed that six participants were more than the 70^th^ percentile compared to age-sex norms based on the Senior Fitness tests [38].

**Table 2:** Baseline Information About Participants

	Median	1	2	3	4	5	6	7	8	9	10
2-min Step Test (steps)	91	129 (90)	71 (25)	186 (90)	97 (75)	118 (90)	121 (90)	84 (50)	73 (50)	45 (10)	63 (25)
Chair Stand Test (reps/30 s)	17	19 (90)	9 (10)	20 (90)	25 (90)	22 (90)	21 (90)	16 (75)	9 (25)	10 (25)	8 (10)
8 foot up and go (feet)	5.7	5.6 (25)	8.4 (10)	5.1 (75)	5.1 (50)	4.9 (75)	5.8 (75)	4.8 (75)	7.9 (25)	9.2 (10)	7.4 (25)
Arm curl (reps /30 s)	15	23 (90)	14 (25)	21 (90)	20 (70)	14 (50)	24 (90)	17 (75)	9 (25)	12 (25)	7 (10)
One leg stance open eyes (sec)	14	15	3	28	14	25	5	45	2	6	45

Physicalfunction scores shown as score (percentile for sex and age norms Rikli & Jones 2013)

Participants self-reported a level of importance (1–10) on 25 barriers to exercise with a maximum score of 250 (Table 3).

**Table 3:** Participant’s Perceived Physical Activity Barriers to Exercise

	***Median***	***1***	***2***	***3***	***4***	***5***	***6***	***7***	***8***	***9***	***10***
Past negative experience	1	1	8	1	1	1	1	1	8	1	2
Lack of time	1	1	1	1	1	7	1	1	6	10	2
Not a priority	6	1	7	1	1	2	1	8	9	10	7
Costs	1	1	1	1	1	8	1	1	5	10	2
Lack of energy	5	1	8	1	5	2	3	4	8	5	6
Lack of knowledge	3	1	3	1	7	2	1	1	9	5	6
Lack of motivation	6	3	8	6	1	9	9	10	5	5	6
Lack of skills	4	2	3	1	3	4	3	6	5	10	6
Uncomfortable in a fitness facility	3	1	10	1	1	2	1	8	9	10	4
Fear of injury	2	1	7	1	1	2	1	1	6	10	3
Fear of an existing condition worse	4	1	6	1	1	5	1	4	6	1	3
How I see my body	3	1	2	7	4	2	3	10	8	1	3
Failure to reach goals in past attempts	2	1	9	1	2	2	2	1	4	1	6
Know that I cannot achieve my goals	2	1	1	1	2	2	3	6	3	1	2
Lack of access to opportunities	2	1	10	1	6	2	1	10	3	1	2
Keep talking myself out of it	2	2	5	1	1	8	8	10	2	1	5
Lack of safe places	1	1	1	1	1	2	1	1	2	1	2
Lack of childcare	1	1	1	1	1	1	1	1	1	1	2
Lack of partner	1	1	1	7	1	1	1	1	2	1	2
Lack of suitable programs	2	1	10	1	2	9	1	10	7	1	5
Lack of support from others	1	1	1	4	2	9	1	1	6	1	2
Lack of transportation	1	1	3	1	1	1	1	1	2	1	2
Hard to keep intensity required	1	1	10	1	10	1	3	1	2	1	5
Peer pressure	1	1	1	1	1	1	3	1	2	1	2
Other	1	1	1	6	1	1	1	1	1	1	1
*TOTAL (___/250)*	**81**	**29**	**118**	**50**	**58**	**86**	**53**	**100**	**121**	**111**	**88**

**Fig. 2:**
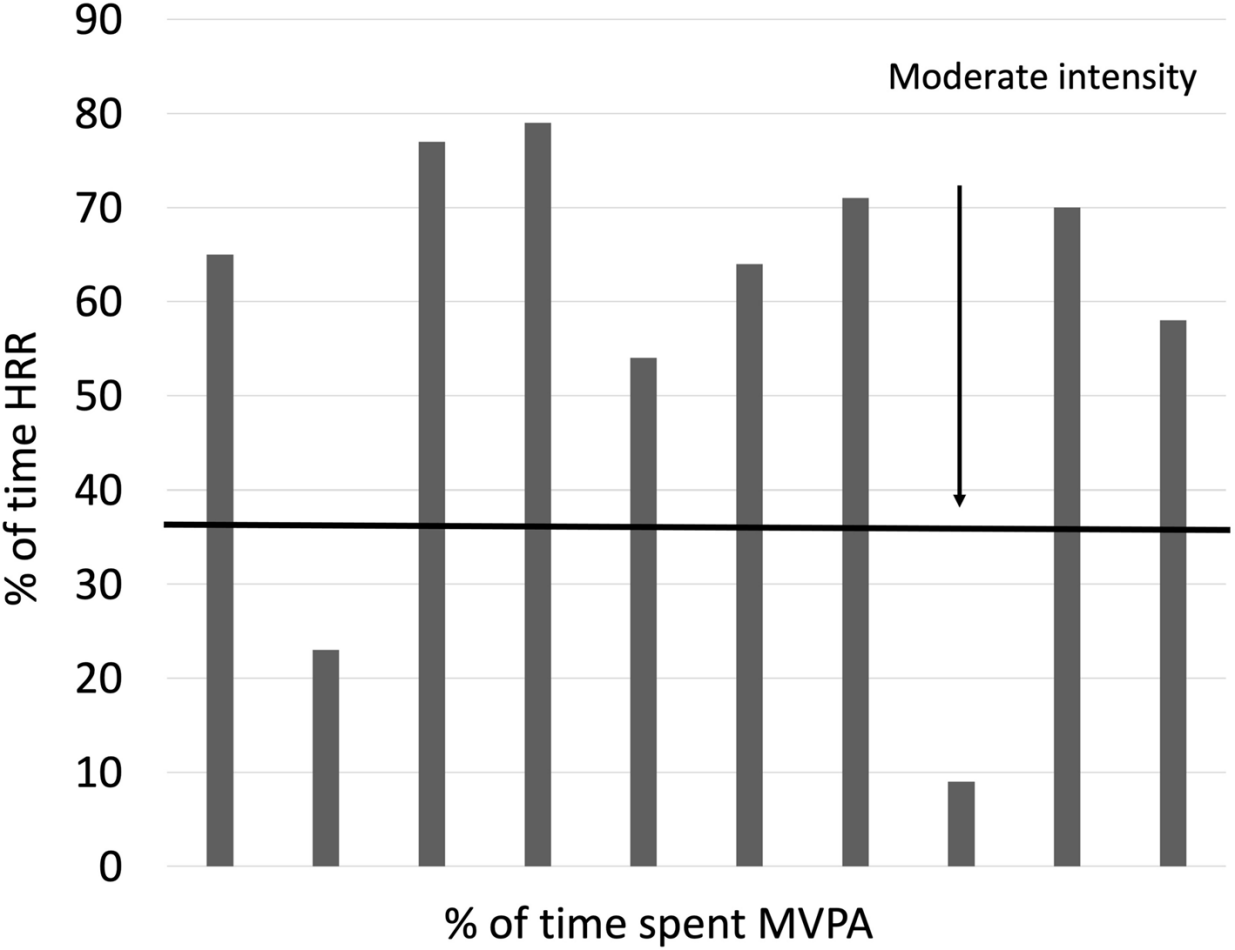
Median Intensity Based on Heart Rate Reserve During a Supervised Session

The most important barriers included: not a priority (6), lack of motivation (6), lack of energy (5), fear of making an existing condition worse (4), lack of skill (4), uncomfortable in a fitness facility (3), and how I see my body (3). Most of the participants increased physical activity when the last week of the intervention was compared to baseline. The median steps/day increased by 12% from 6614 to 7507-steps/day (*p* = 0.02) (Table 4) with 80% of participants increasing their number of daily steps during the intervention. Figure 2 presents the median intensity for each participant when supervised performing the supervised home square stepping exercise pattern at week three. A total of 8 of 10 (80%) of the sample participants reached moderate intensity.

**Table 4:** Daily Steps Per Day

	**Median**	**1**	**2**	**3**	**4**	**5**	**6**	**7**	**8**	**9**	**10**
Baseline	6614	6922	3403	6864	7129	6929	7059	6364	5412	3226	5478
Last week of exercise intervention	7507 *	9175	3674	7297	10,959	10,688	7034	9243	4008	5158	7717

Data are presented as Median from the weekly information

^*^Significantly different from baseline *P* = 0.02

### Qualitative data

Based on charting interview quotes into organized themes (i.e., stage four of Framework Analysis) five barriers to exercise and five enablers activated were identified during participants' experience with the exercise intervention. Table 5 and 6 presents perceived barriers and enablers to the proposed intervention. Actual transcribed quotations are included in these tables. Interestingly, the most important barrier was lack of motivation which may have been modified by participants during the intervention. Participant 1 noted, “10 min is easy, when I first started it felt a little difficult, in the beginning, some days I was at 10 min then I increased it to 15 to 20-min and 10 min, in fact, started to become easy [Achievable Goal].” Participant 8 added, “It is nice, you don’t really need any special equipment. In the hall there’s a treadmill and a bike, at home, I keep my eye on that. But you can just stand up to the wall and do it… [Home equipment].” Finally, Participant 5 stated, “The fact that the program is all set up and it’s not difficult to do on your own [Convenience].”

**Table 5:** Case Examples of Barriers Experienced by Participants

Barrier	Definition	Keywords	Quote
Hard to keep intensity required	Fitness related limitations during exercise program	Fitness, balance, ageing, or fatigue	Participant 2: We live in the country, I want to spend more time outside, but bugs are a problem, the ground is a problem as well. I have a poor sense of balance, I don’t really enjoy walking, and I’m always looking at the ground Participant 1: Well its difficult, it takes a lot of concentration because you have to look down where you want to put your feet. I tried to look up but I had to look down at the boxes…
Lack of time	Conflicts regarding exercise schedule	Too busy, prioritizing activities, unorganized, or travel	Participant 4: See, well, I never reached the total goal [≥ 150-min]. I got 90-min because I did it once a day Participant 2: With difficulty, I had to fit it in my days, day by day I would think ahead, I did it after breakfast and after lunch
Pain	Mild pain during the exercise program	Knee joints, lower back,shoulder joints, leg, or foot	Participant 1: Every time I did the steps for a few minutes I would feel a little pain in the knees as I continued it seemed to go away, my back, lumbar spine gives some pain, occasionally sometimes when I hit the ground and I would feel it in my back Participant 9: I had a difficult time the first week because I did have day surgery on my legs so I had a healing process
Unsuitable program	Dislikes in specific areas of the exercise program	Boring, repetitive, no incentive, no social interaction, too much space	Participant 3: Specifically, I don’t like the home-steps program it’s not for me and it’s too boring just the short distance and rotation… Participant 6: They were simple exercises with the exception of one that I found difficult and that was the triceps exercise and that was only because my back was hurting…
Negative previous experience	Recalls a negative previous experience	Torn tendons in knee, cancer, or injuries from falling	Participant 3: As I told you I busted my ACL tendon. When you do that then you can walk or you can fall, and that’s what actually happened… Participant 2: When I was 50, I did soccer, but my experience was not good, I tore some tendons in my knee really badly

Each quote is an example of a case where the barrier is experienced

**Table 6:** Case Examples of Enablers Experienced by Participants

Enabler	Definition	Keywords	Quote
Achievable goal	Shorter durations became more realistic	Easy/simple to follow, shorter durations	Participant 1: 10 min is easy, when I first started it felt a little difficult, then I increased it to 15 to 20 and 10 min in fact started to become easy Participant 6: Not really, once you keep going you just kind of gain momentum… I always do more than 10 min
Home equipment	Supplied equipment helped physical activity	Helpfulness, convenience, value, flexibility, or no associated costs	Participant 4: Yes, it [i.e., *Home Steps* mat] was always set up… it was a convenience to me I didn’t always have to dig it up Participant 8: It is nice, you don’t really need any special equipment. In the hall there’s a treadmill and a bike, at home, I keep my eye on that. But you can just stand up to the wall and do it…
Convenience	Staying home to complete exercise was convenient	Suitable at home, no cost, own time, simple, no transportation, no gym, no specific attire, weather, short duration	Participant 7: Its convenient too, because you do it at your own time. I don’t like having to be at a certain place at a certain time Participant 5: The fact that the program is all set up and it’s not difficult to do on your own
Fitness	Physical fitness was important	Too inactive, afraid to fall, sedentary, maintaining activity, health benefit/quality of life, stay home longer	Participant 6: Yes, well I realize that I was not doing any exercise, like zero, I knew better but like many people they may have a rational thought about it, but they might not do anything about it Case 2: Weight. My regular routine is sedentary, I do a lot of work at the computer and read books a lot
Preferred home exercise	Prefers exercising at home	Personal space, secure, without communication, indoors, easier to get started, lacking access to facilities	Participant 9:: When you’re home it’s quiet, I have my own place… I enjoyed doing the home steps personally myself Participant 4: I like doing it at home, I wouldn’t go to a gym, I never been to one in my life, I get out and I do a lot around the yard and the house where I live now…

Each quote is an example of a case where the barrier is experienced

The simplicity of the intervention presented a non-daunting task that facilitated participants’ adherence to exercise regardless of the perceived lack of motivation (e.g., overcoming barriers and enabling physical activity). It was simple to do and could be done anywhere in a short amount of time. The provided home equipment, such as the *Home Steps* mat, was also convenient and provided at no cost. Acquiring equipment at little to no cost can have a dramatic effect on physical activity. However, it was important to note that some cases (e.g., Participant 2, Participant 3) thought the *Home Steps* mat required a certain level of concentration described as too much focus needed to perform the exercise.

### Mix-methods

Table 7 provided the framework matrix highlighting cases with high and low total median steps/day with qualitative results. The matrix summarized the connections of self-reported barriers, senior fitness, pre-post total median steps/day, intensity, and themes sorted by row. The first two rows offered a unique representation of cases with the lowest total median steps/day; the last two rows represented cases that reached > 10,000-steps. From the first row, Participant 8 had a low median steps/day and discussed some limitations that were experienced:

Just on the mat, things that came to my mind, just don’t overdo it. Yes, keep a rhythm, but you don’t have to go fast. I think it was going around and around. I was getting dizzy at the end.

**Table 7:** Framework Matrix for Barriers and Enablers

Participant #	Self-reported barriers	Senior fitness	Pre-post steps	Intensity	Themes
Participant 2 Age 74 BMI 30.4 Male	Uncomfortable in fitness facility Access to opportunities Suitable programs Hard to keep intensity	Endurance: 25 Lower strength:10 Agility: 10 Upper strength:25 Balance: 3 s	3403–3674	23%	Prefers home exercise [enabler]: I don’t like changing in front of people, I can’t balance and can’t get my clothes on. I don’t want people watching that… I have had unpleasant experience with changing with people watching and me exercising Hard to keep intensity [barrier]: It's hard, it’s really boring, I was just counting, boring, easier if I had a radio program on, I organized it around radio programs, then it was easy. It's mentally hard when nothing is playing
Participant 8 Age 75 BMI 34.1 Female	Not a priority Knowledge Uncomfortable in fitness facility	Endurance: 50 Lower strength: 25 Agility: 25 Upper strength: 24 Balance: 2 s	5412–4008	9%	Home equipment [enabler]: the materials I used were just great! I didn’t have to buy anything new… Hard to keep intensity [barrier]: Too much at once. If I just cut down and do 5-min and got do my exercises and maybe a week or two later, I add a minute and I can work up to 10 min
Participant 4 Age 70 BMI 32.1 Male	Hard to keep intensity Knowledge Access to opportunities	Endurance: 75 Lower strength: 90 Agility: 50 Upper strength: 70 Balance: 14 s	7129–10,952	79%	Prefers home exercise [enabler]: I like doing it at home, I wouldn’t go to a gym, I never been to one in my life, I get out and I do a lot around the yard and the house… Lack of time [barrier]: If the work hadn’t had come up it wouldn’t had been a big deal… this would’ve been fun
Participant 5 Age 67 BMI 33.1 Female	Social support Suitable programs Motivation Cost Self-talk	Endurance: 90 Lower strength: 90 Agility: 75 Upper strength: 50 Balance: 25 s	6921–10,688	54%	Convenience [enabler]: The fact that the program is all set up and it's not difficult to do on your own… we’ve got the thing [Home Steps mat], we have time, we have Alexis [AI device] for a timer… we had a backpack with a weight in it… Pain [barrier]: When I was younger, I use to go to the gym all the time, step classes etc., but now you know bad knees and feet or back and now you don’t do those kinds

Intensity reaching MVPA at 40% and more

Participant 2 had a low number of steps/day and had difficulty balancing. They also perceived a barrier total of 118/250 based on the level of importance. Participant 2 elaborated on a negative previous experience in their 50’s while playing soccer when they tore some tendons in their knee:

I do a lot of work at the computer and read books a lot. We live in the country, I want to spend more time outside, but the bugs are a problem. We have a treadmill at home to have me do exercise, if I’m listening to a food radio program or watching TV programs. I have a lot of reasons I come up with for not doing exercise. That’s why I wanted to come here to be more active in the home without involving much equipment.

To reduce data, the framework matrix summarized information on Participant 2 and 8 who reported a low median number of steps/day, and Participant 4 and 5, who had a median number of steps/day > 10,000 steps/day, to compare, contrast and search for patterns. Participants having a lower physical activity level were reaching lower intensity when performing the square stepping exercise during a supervised session (23% and 9% vs. 79% and 54%. The barriers however by these two groups were not distinct with some reported by participants with lower and higher physical activity levels. For example, having a suitable program, hard to keep the intensity were barriers reported by participants in both spectrums of physical activity level.

## Discussion

The results show that square stepping exercises targeted some barriers and enablers and could lead to more regular physical activity at moderate intensity. Home square stepping exercises could be more suited for some than others; inactive older adults who are intimidated by a fitness facility setting and concerned with their body image. Stokols (1996), stated that, “Efforts to modify individuals’ unhealthy behaviors and lifestyles have been guided by several distinct theories of social influence” (p.283). As such, to understand the barriers and enablers associated with our findings we used the socioecological model. Stokols (1996) work explains that socioecological models are foundationally rooted in the core principles of “interrelations among environmental conditions and human behavior and well-being” (p. 285).

### Barriers

The commonly reported barriers for older adults to be regularly active are lack of time, pain/injury, lack of social support, poor health/illness, and lack of knowledge [7–9, 12]; are mostly at the individual and interpersonal levels of the socioecological model [46]. The barriers were not observed to be of high importance in this sample when self-reporting perceived barriers to exercise at baseline. This could be in part related to the characteristics of the sample since most of the participants were highly functional, highly educated, and financially comfortable. However, the five most reported barriers (i.e., not a priority, lack of motivation, lack of energy, fear of making an existing condition worse, lack of skill, uncomfortable in a fitness facility, and how I see my body) were also at the individual and interpersonal levels of the sociological model. It is possible that a future intervention using the SSE could target organizational, community, and policy levels in addition to the other two elements as an attempt to have a greater odds to impact the adherence to physical activity [47].

Motivation to exercise, also an individual element of the sociological model, could be linked to other barriers; for example, the perception of lack of time was often related to a lack of motivation to exercise [48]. A lack of time was reported by the participants of this study. The perception of a lack of time and a lack of motivation are both modifiable [48]. Motivation is dependent on self-discipline when trying to adhere to an exercise program [9]. However, motivation is a multi-dimensional construct suggesting that a host of barriers could be linked to not feeling like exercising. There were many reported barriers in the literature; however, none of the participants in the literature overcame all barriers. Participants who completed home square stepping exercise intervention did not overcome all barriers listed prior to the intervention but overcame commonly reported barriers, mostly those related to the individual elements of the socioecological model since the program was made to be done alone in participants’ home.

Barriers reported at baseline were not always reported in the interviews. This showed that perceived barriers are dynamic and depend on the question and the environment in which the activity is offered. The participants reported barriers to exercise at baseline and even if they overcame some of these, participants identified other barriers after the intervention. This phenomenon can be explained by the fact that habits and behaviour patterns change [49]. It is possible that interpersonal, organizational, and community barriers /enablers could have been reported if *Home Steps* had been offered in a common space and in groups. For example, participants who performed *Home Steps* did not experience constraints with accessibility and the feeling of being uncomfortable in a fitness facility. Therefore, the context in which the activity is delivered can tackle different elements of the socioecological model. Not only barriers can change based on the context but also changes over time for the same individual.

### Enablers

The most common enablers to regular exercise are barriers, mostly related to individual and interpersonal levels of the socioecological model [46]. This could be due to the idea that individuals are responsible of their choices and behaviour even if the social environment is associated with participation in physical activity level [50]. Effective exercise for older adults can stimulate exercise enablers such as the perception of health benefits at the individual and interpersonal levels such as an improvement in balance, toning, cardiovascular health social support, and enjoyment [8–13]. In the current study, one of the enablers found in the interviews was increasing fitness which is similar to the literature around wanting to improve health [8] by increasing their baseline physical activity level; an enabler at the individual level if the socioecological model.

Most participants completed the proposed home square stepping exercise intervention and reached moderate intensity when supervised. However, based on the interviews, the proposed exercise may not be the preferred methods for adults aged 75 and older to reach moderate intensity as they reported more barriers to keep moderate intensity because of a lack of energy or motivation. This is consistent with some barriers in the literature suggested for this demographic [8]. Even if some people can’t or don’t have the desire to reach moderate intensity while performing the square stepping exercises it is important to note that many benefits (e.g., lower waist circumference and lower triglyceride level [51]) are associated with light physical activity compared with no activity [52].

### Physical activity level

The number of steps that increased to the recommended level of a minimum of 7000 steps/day for older adults could be due to the high baseline of the participants’ functional abilities and the short-term program [27], however, only two participants reached the 10,000 goal for adults of all ages [27]. It is important to note that this short-term intervention resulted in acute effects and long-term effects of any program normally decline over time. Long-term follow-up of these participants would be important to see the true value of the intervention to enhance physical activity levels. Another study have shown a significant increase in physical activity level, for people living with Multiple Sclerosis using the stepping-square exercise mat over a period of 12 weeks [53]. In addition, it is important to note that even if only two participants reached an average 10,000 steps per day at the end of this short intervention, 80% of participants increase their steps per day. The latest physical activity guidelines in Canada do recognize that any movement can lead to a reduction in mortality rate [52].

### Limitations

This study is not without limitations such as only using a small sample size for increasing physical activity over a short period of three weeks. The use of home square stepping exercises was also not tracked at home, so it is impossible to know if participants reached the recommended sessions at home and the intensity reached during those sessions. Concerns were raised within the ethics board when entering participant homes was suggested. It was suggested to conduct an in-person session mimicking the home setting as an alternative. Participants seemingly enjoyed the visits with the research staff present, so this could have developed a bias towards encouraging participation, increasing adherence to the intervention, and enjoying visits rather than the square stepping exercise. This study used the framework approach for analysis which was overseen by an experienced researcher; however, the framework approach required similar research objectives. The majority of participants had post-secondary education and a high-income status which is not the norm for the target population. This could have been caused by the location of data collection, the university setting, or the strategy used to attract participants (e.g., internet). The demographics of the sample may have been an underlining foundation of the themes that emerged for barriers at baseline, barriers overcame during the intervention, and activated enablers.

Future studies should aim to demonstrate long-term feasibility of Home Steps. Overall, the proposed Home-Step exercise program could potentially increase physical activity level at the recommended intensity by reducing barriers to exercise and enhancing enablers, but a longer supervised intervention with an adequate sample size would be required.

## Impact/Conclusion

The proposed home square stepping exercise intervention shows that this strategy could increase the physical activity level of older adults considered inactive, overcome barriers, and activated enablers to exercise, especially individual and interpersonal barriers. Older inactive adults who are uncomfortable in a fitness facility or those who do not have a great perception of their body image could potentially overcome these barriers from the square steps exercise intervention. Home square stepping exercises could be a suitable exercise strategy for older adults to increase the amount of time spent at light and moderate intensity.

## Supplementary information

**Additional file 1.** Semi-Structured Interview Guide. This is the interview guide that the interviewer used to guide their interview and make sure that specific questions were asked. For this study, the literature review yielded general semi-structured questions which were pilot tested. To choose appropriate questions for the interview guide, two older adult volunteer participants in the community completed at 30–60-min interview. Upon completion of the interview process, questions that generated: ‘yes’ or ‘no’ responses, difficulty in understanding, and a negative response (i.e., feeling uncomfortable because it felt personal), were modified to make it more open-ended and generate effective dialogue. The final interview guide was reviewed by a member of the research team and it focused on areas that included: reason for participating, exercise elements, satisfaction, and overall safety.

## Data Availability

The datasets used and/or analysed during the current study are available from the corresponding author on reasonable request.

## Abbreviations

WHO: World Health Organization
CSEP: Canadian Society for Exercise Physiology
RHR: Resting Heart Rate
BP: Blood Pressure
SFT: Senior Fitness Test
MVPA: Moderate to Vigorous Physical Activity
HR: Heart rate
BMI: Body mass index
HRR: HR reserve
