# Supplementary material for: Potential value of home square-stepping exercises for inactive older adults: an exploratory case study
Source: BMC Geriatr. 2022 Jan 3;22:14. doi: 10.1186/s12877-021-02712-x (PMC8722225; doi:10.1186/s12877-021-02712-x)
Supplement: Supplementary file 1 — Additional file 1. Semi-Structured Interview Guide. This is the interview guide that the interviewer used to guide their interview and make sure that specific questions were asked. For this study, the literature review yielded general semi-structured questions which were pilot tested. To choose appropriate questions for the interview guide, two older adult volunteer participants in the community completed at 30–60-min interview. Upon completion of the interview process, questions that generated: ‘yes’ or ‘no’ responses, difficulty in understanding, and a negative response (i.e., feeling uncomfortable because it felt personal), were modified to make it more open-ended and generate effective dialogue. The final interview guide was reviewed by a member of the research team and it focused on areas that included: reason for participating, exercise elements, satisfaction, and overall safety. [file 12877_2021_2712_MOESM1_ESM.pdf]

Semi-Structured Interview Guide

Participating

1. Can you please explain your reasons for increasing your physical activity level?
   Probe: Describe what being physically active means to you?

Exercise Program

2. What made you come back to the Home Steps Program for all visits?
   Probe: Would you require visits to continue Home Steps?
3. What role would you say phone calls played for you during the week with no visit?
   Probe: Would you require communication to continue Home Steps?
4. Can you explain the Home Steps weekly goal to someone interested in knowing more about the program? Is the square-stepping exercise new to you?
   Probe: What made it easy or hard to understand?
5. Was maintaining 10-minutes of square-stepping exercise hard or easy?
   Probe: What helped you continue after 10-minutes? What stopped you from reaching 10-minutes and more? What feelings come to mind about square-stepping exercise?
6. Can you describe how you scheduled reaching the weekly goal to accumulate your minutes?
   Probe: What things made exercising at home easier? What things made exercising at home harder?
7. Can you tell me about your experience with exercise protocol?

Satisfaction

8. Are there benefits to your overall physical activity with completing the Home Steps Program? Did you combine other exercises?
   Probe: What do you like/enjoy about exercising at home?
9. Are there drawbacks to your overall physical activity with completing the Home Steps Program? Did it make you stop other activities?
   Probe: What do you dislike/hate about exercising at home?
10. To what extent did the materials you used at home help or hinder your ability to complete the Home Steps Program?
    Probe: Explain how you would use materials in your home for long-term exercise? Would you try being more creative with your materials?

Safety

11. Did you feel you were going to fall at any point in the Home Steps program?
    Probe: Can you recall a time and please describe the moment with as much detail as possible?
12. Did you experience any pain/injury at any point in the Home Steps Program?
    Probe: Can you recall a time and please describe the moment with as much detail as possible?
13. On a scale of 1-10 how comfortable are you completing the Home Steps program to reach the weekly goal?
14. Do you have anything you want to add?
